# Intraoperative Molecular Imaging Can Detect Large Nerve Perineural Invasion: A Case Report

**DOI:** 10.1002/hed.70160

**Published:** 2026-01-07

**Authors:** Carleigh R. Burns, Aviva S. Mattingly, Kim Ely, Andreja Radevic, Nicole Meeks, Sydea Maria Ahmad Zaidi, Brandee Brown, Georgii Vasiukov, Michael Topf, Eben Rosenthal

**Affiliations:** ^1^ Department of Otolaryngology – Head and Neck Surgery Vanderbilt University Medical Center Nashville Tennessee USA; ^2^ Department of Pathology, Microbiology and Immunology Vanderbilt University Medical Center Nashville Tennessee USA

**Keywords:** fluorescence‐guided surgery, head and neck squamous cell carcinoma, intraoperative visualization, Panitumumab‐IRDye800, perineural invasion

## Abstract

**Background:**

Perineural invasion (PNI) in head and neck squamous cell carcinoma (SCC) results in worse overall survival. Diagnosis requires resection and microscopic evaluation.

**Methods:**

A 63‐year‐old male with persistent cT4aN0 p16‐positive SCC of the left base of tongue following chemoradiotherapy underwent salvage total glossectomy. Fluorescence‐guided imaging of the wound bed was performed with PDE‐GEN3 near‐infrared (NIR) imaging following infusion of an optically EGFR‐targeted antibody, Panitumumab‐IRDye800 (pan800).

**Results:**

The proximal resected hypoglossal nerve was imaged intraoperatively and demonstrated a strong green fluorescence signal, raising concern for subclinical PNI. Biopsy of the nerve revealed SCC on frozen section analysis. This was re‐resected with subsequent proximal margin negative for carcinoma. Postoperatively, ex vivo imaging of the nerve using PDE‐GEN3 NIR imaging again demonstrated the presence of pan800 within the initial positive nerve margin, confirming subclinical PNI.

**Conclusions:**

This case shows the feasibility of intraoperative fluorescence as a method to help identify subclinical PNI.

**Trial Registration:** ClinicalTrials.gov identifier: NCT05945875

## 1 Introduction

Perineural invasion (PNI) is a histologic growth pattern where tumor cells grow alongside or within a nerve. PNI frequently occurs in head and neck squamous cell carcinoma (HNSCC) and is associated with poor overall survival, increased risk of recurrent disease, and a high likelihood of metastasis [1, 2, 3]. Additionally, PNI influences the need for adjuvant radiation therapy (RT), which also has significant treatment‐related morbidity [4].

PNI is a separate entity from perineural spread (PNS), which is gross nerve involvement and is frequently visible on preoperative imaging [5]. However, magnetic resonance imaging (MRI) and computed tomography (CT) typically fail to identify PNI in clinically asymptomatic patients [1]. Therefore, the only intraoperative diagnostics for PNI detection are methods such as frozen section analysis (FSA), where suspicious nerves are sent to pathology for microscopic evaluation [6]. Because the decision to send intraoperative frozen sections of nearby nerves is surgeon‐dependent and not always performed, successful identification of subclinical PNI during the resection remains an unmet clinical need with the potential to spare the patient additional oncologic intervention, including re‐excision and/or adjuvant therapy.

Previous studies have demonstrated the utility of intraoperative margin visualization through fluorescence‐guided surgery (FGS), which allows for real‐time detection of positive margins, acting as a visual guide for the surgeon [7, 8]. Our team has previously investigated Panitumumab‐IRDye800 (pan800), an epidermal growth factor receptor (EGFR) targeted antibody that allows for near‐infrared (NIR) fluorescence imaging. These fluorescently labeled antibodies can be utilized for real‐time evaluation of margins, identification of potential secondary primary lesions, and to guide sampling of intraoperative frozen sections [8]. Despite this prior research, we have not evaluated the potential utilization of this method to help identify PNI.

In this study, we present a case of subclinical hypoglossal nerve PNI detected using intraoperative fluorescence, guiding immediate re‐resection with final negative margins. This case emphasizes the potential for FGS to successfully detect PNI in real time and its potential role in guiding head and neck cancer surgery.

## 2 Case Presentation

A 63‐year‐old male with a history of cT4aN0 p16‐positive SCC of the base of tongue who completed definitive chemoradiation therapy 4 months prior was referred to our institution with concern for persistent disease. Following completion of CRT, he had worsening tongue pain radiating to the left ear. A contrast‐enhanced CT scan of the neck revealed persistent enhancement of the left base of tongue mass extending across midline with no cervical adenopathy. Post‐treatment positron emission tomography‐CT (PET‐CT) scan showed continued fluorodeoxyglucose (FDG) uptake along the left BOT SUV of 8.1, with no FDG‐avid cervical lymphadenopathy. Due to these findings, the patient was scheduled for a repeat DL with biopsy which was positive for persistent squamous cell carcinoma (SCC). The patient was enrolled in a clinical trial (NCT05945875) using an optically EGFR targeted antibody, pan800, which was infused 6 days prior to surgery. He subsequently underwent total glossectomy, left neck dissection, and anterolateral thigh free flap reconstruction.

**FIGURE 1:**
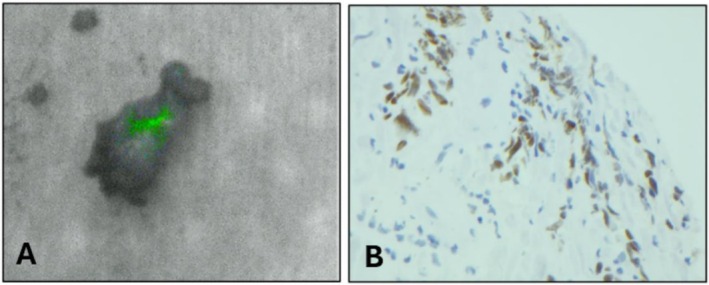
(A) Frozen section biopsy of hypoglossal nerve imaged on back table with PDE‐GEN3 NIR prior to pathology processing demonstrating a green fluorescent signal. (B) p40 immunostain used to highlight squamous cells showing evidence of carcinoma.

**FIGURE 2:**
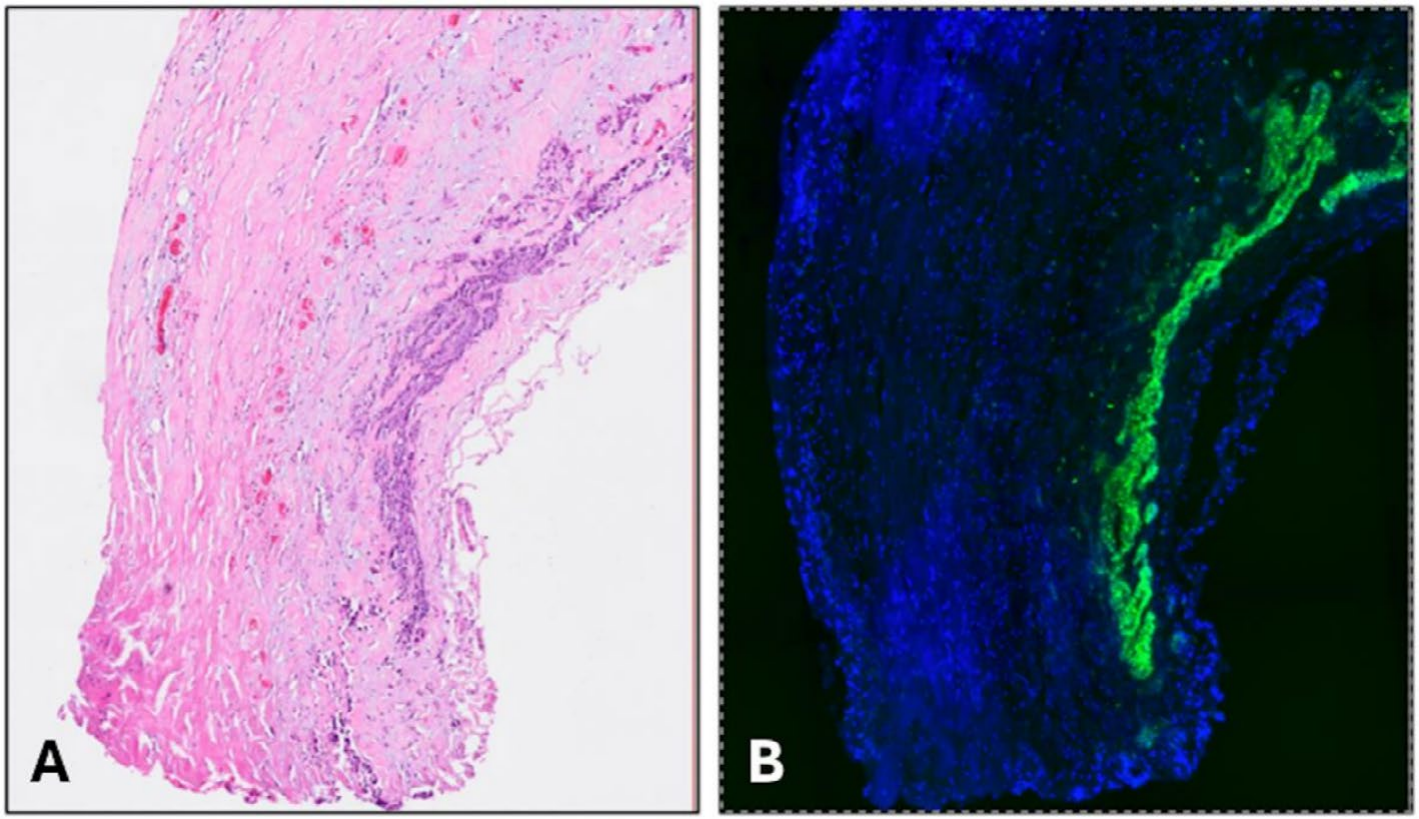
(A) Ex vivo Hematoxylin and Eosin (H&E) slide of resected proximal hypoglossal nerve demonstrating squamous cell carcinoma. (B) Immunofluorescence imaging displaying the presence of carcinoma and corresponding pan800 within the initial positive hypoglossal nerve margin.

Following resection, the PDE‐GEN3 NIR imaging system (Hamamatsu Photonics K.K., Hamamatsu, Japan) was brought into the field to visualize real‐time pan800 fluorescence of the wound bed. The proximal left hypoglossal nerve displayed a strong focus of green fluorescence signal compared to the background tissue as well as the nearby spinal accessory nerve (Figure 1). This raised concern for potential subclinical PNI and guided the surgeon to biopsy the proximal left hypoglossal nerve, which was positive for SCC on FSA. Re‐resection of the proximal hypoglossal nerve was immediately performed intraoperatively, and a subsequent proximal margin was negative for carcinoma. Postoperatively, the resected proximal hypoglossal nerve was further imaged ex vivo using PDE‐GEN3 near‐NIR imaging, which confirmed the presence of pan800 within the initial positive nerve margin. Final pathology revealed a 3.3 cm p16‐positive SCC and confirmed the intraoperative proximal hypoglossal nerve SCC (Figure 2).

## 3 Discussion

We report the use of intraoperative immunofluorescent imaging of the proximal hypoglossal nerve identified sub‐clinical PNI and guided re‐resection to negative margins. To our knowledge, this is the first case that utilized FGS in real time to intraoperatively identify subclinical PNI. Our prior studies in FGS demonstrated value for resection of the primary tumor margins and metastatic lymph nodes [8, 9]. Another study described the use of Dynamic Optical Contrast Imaging (DOCI) to detect PNI in an in vivo murine model, being able to detect microscopic PNI with 100% sensitivity and specificity [10]. While its use in humans was not evaluated, this preclinical study demonstrates that there is considerable potential to improve intraoperative detection of PNI and thus improve intraoperative decision making.

Importantly, in this case the hypoglossal nerve had already been resected, and in vivo imaging revealed a strong fluorescence signal that prompted re‐resection, confirming additional carcinoma. Whether fluorescence alone should prompt resection of an otherwise intact cranial nerve has not been explored, requires further investigation, and is not recommended by the authors. As a proof‐of‐concept, this case establishes the feasibility of FGS to detect PNI and guide intraoperative decision making in real time. However, we present a single use case and would require a larger cohort of HNSCC oncologic resection cases and randomization to validate its use and generalizability. There is potential for this to be an effective visualization mechanism during oncologic surgical resections moving forward.

## 4 Conclusion

In this case, intraoperative visualization using FGS allowed for subclinical hypoglossal nerve PNI detection in real time, guiding immediate reresection and final negative margins. Further studies reporting the effectiveness and significance will be required to uncover the viability of this technique.

## Author Contributions

Carleigh Burns: conceptualization, data curation, visualization, writing – original draft preparation, writing – review and editing. Aviva S. Mattingly: conceptualization, data curation, methodology, writing – review and editing. Kim Ely: pathological analysis, validation, writing – review and editing. Andreja Radevic: data curation, visualization, writing – review and editing. Nicole Meeks: clinical investigation, data acquisition, writing – review and editing. Sydea Maria Ahmad Zaidi: data analysis, writing – review and editing. Brandee Brown: project administration, data management, writing – review and editing. Georgii Vasiukov: methodology, validation, writing – review and editing. Michael Topf: supervision, conceptualization, methodology, writing – review and editing. Eben Rosenthal: supervision, conceptualization, writing – review and editing.

## Funding

This work was supported by a National Cancer Institute (NCI) K08 Career Development Award (5K08CA293255‐02).

## Ethics Statement

This study was conducted under an Institutional Review Board‐approved protocol (IRB#230327) at Vanderbilt University Medical Center.

## Consent

Written informed consent was obtained from the patient for publication of this case and its accompanying images.

## Conflicts of Interest

The authors declare no conflicts of interest.

## Data Availability

The data that support the findings of this study are available from the corresponding author upon reasonable request.

## References

[1] S. Purohit , P. Ahlawat , S. Tandon , A. Jain , and M. Gairola , “Challenges Seen With Peri‐Neural Invasion in Head and Neck Cancer – A Review Article,” Oral Oncology Reports 6 (2023): 100028, 10.1016/j.oor.2023.100028.

[2] Z. Y. Tao , G. Chu , and Y. X. Su , “The Prognostic Role of Perineural Invasion for Survival in Head and Neck Squamous Cell Carcinoma: A Systematic Review and Meta‐Analysis,” Cancers (Basel) 16, no. 14 (2024): 1–2, 10.3390/cancers16142514. PMC11274576 39061154

[3] L. B. Schmitd , C. S. Scanlon , and N. J. D'Silva , “Perineural Invasion in Head and Neck Cancer,” Journal of Dental Research 97, no. 7 (2018): 742–750, 10.1177/0022034518756297. 29443582 PMC6728584

[4] S. Ghosh Laskar , A. Kumar , A. Adhau , et al., “Rethinking Radiation Therapy for Perineural Invasion in Oral Cancer: Does More Coverage Improve Outcomes?,” Cancer Medicine 14, no. 15 (2025): e71134, 10.1002/cam4.71134. 40792421 PMC12340603

[5] J. Y. Tong , S. C. Huilgol , C. James , S. Rajak , and D. Selva , “Perineural Invasion and Perineural Spread in Periocular Squamous Cell Carcinoma,” Eye (London, England) 37, no. 5 (2023): 875–884, 10.1038/s41433-022-02306-w. 36400852 PMC10050156

[6] M. L. Urken , J. Yun , M. P. Saturno , et al., “Frozen Section Analysis in Head and Neck Surgical Pathology: A Narrative Review of the Past, Present, and Future of Intraoperative Pathologic Consultation,” Oral Oncology 143 (2023): 1–2, 10.1016/j.oraloncology.2023.106445. 37285683

[7] H. W. White , A. B. Naveed , B. R. Campbell , et al., “Infrared Fluorescence‐Guided Surgery for Tumor and Metastatic Lymph Node Detection in Head and Neck Cancer,” Radiology: Imaging Cancer 6, no. 4 (2024): e230178, 10.1148/rycan.230178. 38940689 PMC11287229

[8] S. van Keulen , N. Nishio , S. Fakurnejad , et al., “The Clinical Application of Fluorescence‐Guided Surgery in Head and Neck Cancer,” Journal of Nuclear Medicine 60, no. 6 (2019): 758–763, 10.2967/jnumed.118.222810. 30733319 PMC6581234

[9] N. Meeks , S. James , G. Krishnan , et al., “Background Tissue With Native Target Expression Can Determine Presence of Nodal Metastasis in Head and Neck Squamous Cell Carcinoma Patients Infused With Targeted Fluorescent Tracers,” Molecular Imaging and Biology 27, no. 3 (2025): 333–340, 10.1007/s11307-025-01996-4. 40100567 PMC12162778

[10] K. Tam , Y. Alhiyari , S. Huang , et al., “Label‐Free, Real‐Time Detection of Perineural Invasion and Cancer Margins in a Murine Model of Head and Neck Cancer Surgery,” Scientific Reports 12, no. 1 (2022): 12871, 10.1038/s41598-022-16975-w. 35896579 PMC9329308

